# Activity-Based Protein Profiling Identifies an α-Amylase Family Protein Contributing to the Virulence of Methicillin-Resistant *Staphylococcus aureus*

**DOI:** 10.1021/acsinfecdis.4c00638

**Published:** 2025-02-07

**Authors:** Md Jalal Uddin, Kjersti Julin, Herman S. Overkleeft, Mona Johannessen, Christian S. Lentz

**Affiliations:** † Centre for New Antibacterial Strategies (CANS) and Research Group for Host-Microbe Interactions, Department of Medical Biology (IMB), UiT—The Arctic University of Norway, 9019 Tromsø, Norway; ‡ Leiden Institute of Chemistry, Leiden University, Einsteinweg 55, 2333 CC, Leiden, The Netherlands

**Keywords:** chemoproteomics, activity-based probe, glycoside hydrolases, biofilm, virulence

## Abstract

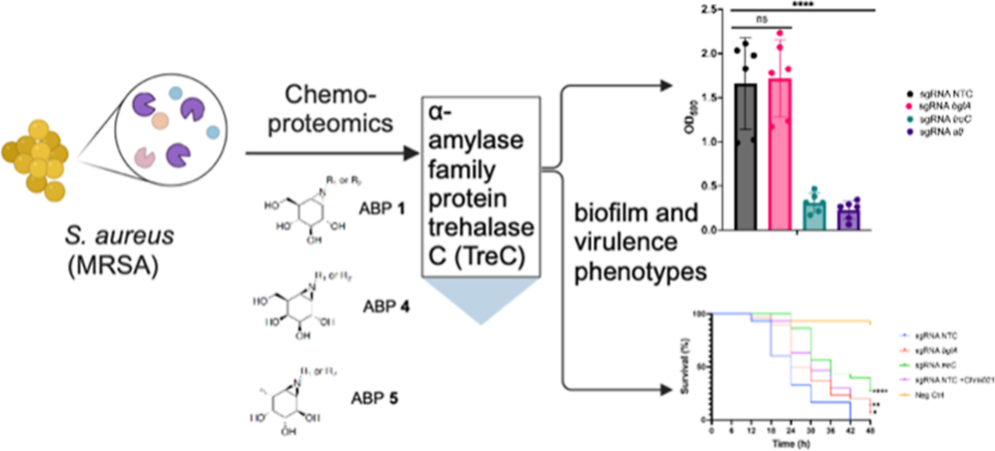

In search of new putative antimicrobial drug targets in methicillin-resistant *Staphylococcus aureus*, we aimed to identify and characterize retaining glycosidase activities in this bacterial pathogen. Using activity-based protein profiling (ABPP), a panel of 7 fluorescent probes was screened to detect activities of diverse retaining glycosidase families. Based on this, a cocktail of 3 biotinylated probes (targeting α-glucosidases, β-galactosidases and α-fucosidases) was used for target enrichment and three glycoside hydrolase family proteins were identified by mass-spectrometry: 6-phospho-β-glucosidase (BglA), α-amylase family protein trehalase C (TreC), and autolysin (Atl). The physiological relevance of previously uncharacterized BglA and TreC was addressed in CRISPRi and inhibitor studies with the putative TreC inhibitor α-cyclophellitol-aziridine. Silencing of *tre*C did not affect bacterial growth in rich media, but reduced biofilm formation *in vitro*, and attenuated virulence during *Galleria mellonella* infection, warranting future investigations into the biochemical function of this enzyme.

*Staphylococcus aureus* is a Gram-positive opportunistic pathogen that colonizes the nose and/or pharynx, skin, and intestine in approximately one-third of the human adult populations. Facilitated by its unique ability to adapt to diverse environments, manipulate host cells, and evade the immune system,^1^*S. aureus* causes a range of infections, from mild skin infections to life-threatening diseases, including bacteremia, endocarditis, osteomyelitis, and device-related infections.^2−5^ Antibiotic resistant strains such as methicillin-resistant *S. aureus* (MRSA) are spreading rapidly and contribute significantly to the unfolding of the antimicrobial resistance crisis, urging the development of novel treatment options.^6^

One putative class of drug target enzymes that has not been systematically explored in *S. aureus* are glycoside hydrolases (GH, also termed as glycosidases). By catalyzing the cleavage of glycosidic bonds, GHs fulfill important functions in all life forms, and some are considered as drug targets for diabetes mellitus type 2 and cancer (reviewed in^7,8^) or as biomarkers for precision medicine.^9^ Recently, also enzymes involved in the degradation of peptidoglycan are gaining interest as drug targets. *S. aureus* cell envelope consists of a variety of glycan structures including peptidoglycan (murein), capsule polysaccharide, and teichoic acids. Enzymes involved in the biosynthesis of peptidoglycan such as the penicillin-binding proteins are targeted by β-lactam antibiotics. Autolysin is a *S. aureus* peptidoglycan hydrolase with both *N*-acetylmuramyl-*l*-alanine amidase and endo-β-*N*-acetylglucosaminidase activity.^10^ This enzyme plays a role in cell envelope remodeling, virulence, and biofilm formation^11^ and is the target of some recently discovered type V glycopeptide antibiotics.^12,13^ Recent studies have also demonstrated the importance of wall teichoic acid (WTA) glycopolymers for the interactions with host epithelial cells.^14^ Specific glycosylation patterns of WTA on the *S. aureus* surface affect recognition recognized by human Langerin cells^15^ and affect their susceptibility to vancomycin.^16^ Enzymes that would dynamically cleave and modulate these glycosides have to the best of our knowledge not been reported to date. In addition, bacterial pathogens are continuously exposed to host-derived glycans which they could cleave for use as a nutrient source, to promote invasion or immune evasion.^17−19^ We therefore hypothesized that *S. aureus* GHs with putative roles in cleaving endogenous or exogenous glycans remain uncharacterized.

Activity-based protein profiling (ABPP) uses functionalized covalent and irreversible inhibitors (called activity-based probes, ABPs) to detect, enrich, and identify active species of an enzyme family of interest in a biological sample. Previously, we have utilized fluorophosphonate probes^20^ and carmofur-derived ABPs^21^ to identify uncharacterized serine hydrolases in *S. aureus*, and we have demonstrated a role of the newly identified fluorophosphonate-binding hydrolase B, FphB in virulence^20^ and suggest a role of FphH in bacterial stress response.^22^ We have been seeking to expand these studies to other enzyme families. Retaining GHs cleave their substrate using a mechanism that involves a covalent glycosyl-enzyme intermediate,^23^ and they are inhibited covalently and irreversibly by cyclophellitols/cyclophellitol aziridines emulating the configuration of the parent substrate glycoside. Analogs of these inhibitors functionalized with a fluorophore, biotin or click-handle serve as covalent ABPs for detection of active GHs.^24−26^ GHs generally have a high degree of substrate selectivity and act on either α- or β-glycosidic bonds, discriminate between configurational isomers and differ in their preference of cleaving bonds from the nonreducing end (exoglycosidases) or within oligo-polysaccharide chains (endoglycosidases).^27^ Consequently, there is no universal broad-spectrum probe for retaining GHs with diverse substrate specificities. However, a diverse panel of GH probes has been developed to target α- and β-GHs with diverse substrate specificities and allowing for targeting both retaining exo- and endo-GHs.^28,29^ These probes have been instrumental in detecting GHs enzymes in human cells/tissues^30,31^ plants,^32^ and microbes.^9,33−37^

In this study, we set out to identify active GHs in *S. aureus* using ABPP. We initially screened a panel of fluorescent GH-ABPs to detect active GHs in *S. aureus* under diverse growth conditions. We continued to use a combination of three pooled biotinylated probes for target enrichment and identification by mass spectrometry. We report the identification of two previously uncharacterized enzymes, namely, putative 6-phospho-β-glucosidase (BglA) and the α-amylase family protein trehalase C (TreC). The functional relevance of these enzymes was investigated during *in vitro* growth in rich media, in biofilm formation and by *in vivo* infection of *Galleria mellonella* larvae using CRISPRi knock-downs strains and studies with a putative TreC inhibitor. Our data support a role of TreC in biofilm formation and virulence during *G. mellonella* infection, whereas BglA only had minor relevance in the infection model.

## Results and Discussion

### Gel-Based Activity-Based Protein Profiling Using Fluorescent Glycosidase ABPs

**Scheme 1:**
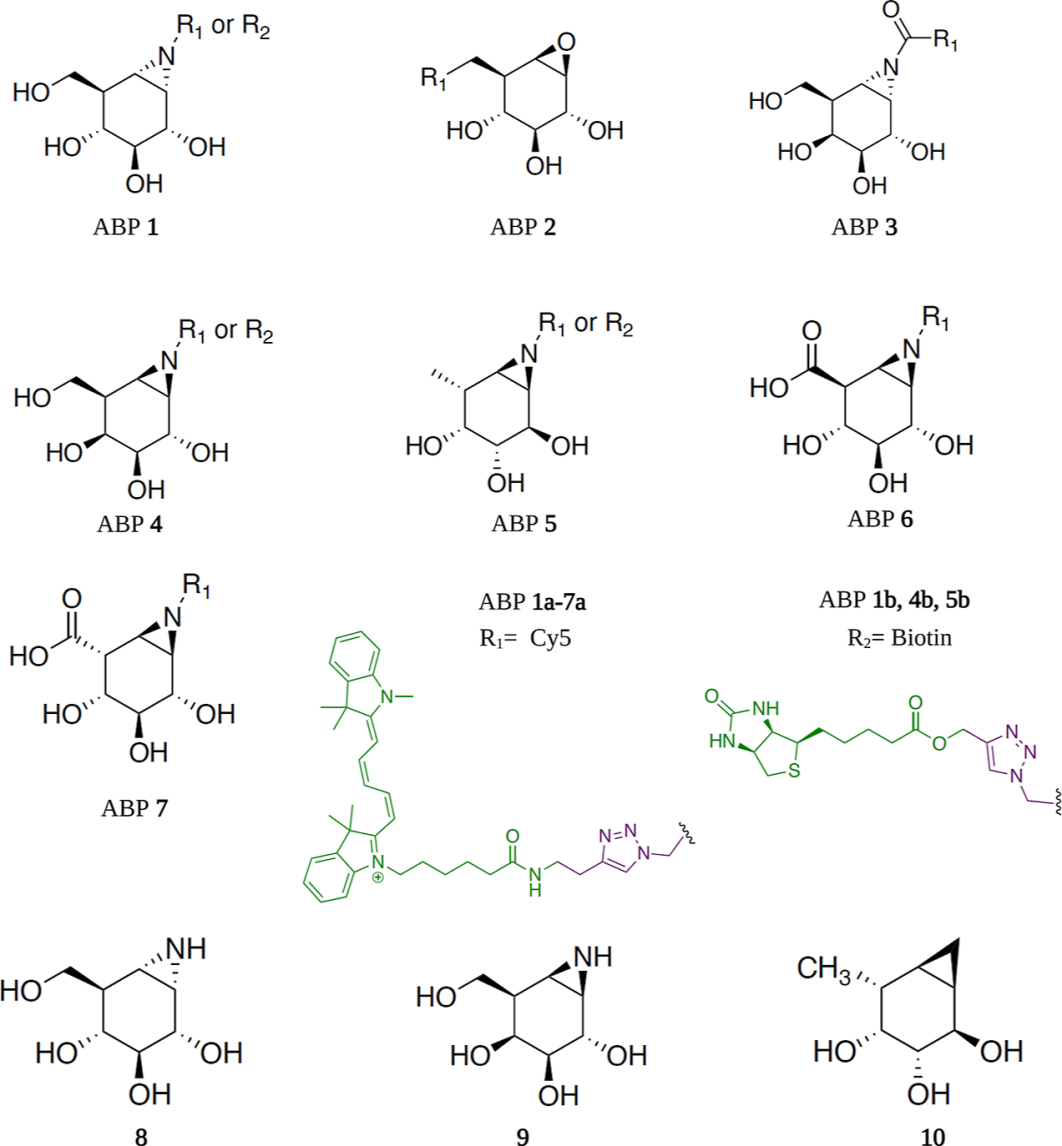
Chemical Structures of GH-Targeting Activity-Based Probes Used in This Study ABP **1** (α-glucosidases), ABP **2** (β-glucosidase), ABP **3** (α-galactosidase), ABP **4** (β-galactosidase), ABP **5** (α-fucosidases), ABP **6** (β-glucuronidase), ABP **7** (α-iduronidases), each equipped with functional groups Cy5 (*R*_1_) (probe **1a**–**7a**) or biotin (*R*_2_) (probe **1b**, **4b**, **5b**). Structures of parent inhibitors **8** (Chris021), **9** (TB562), **10** (JJB330).

To profile retaining GH activities in *S. aureus* we initially used a panel of fluorescent ABPs developed previously to selectively target α-glucosidases^38^ (ABP **1a**), β-glucosidases^39^ (ABP **2a**), α-galactosidases^40^ (ABP **3a**), β-galactosidases^41^ (ABP **4a**), α-fucosidases^42,43^ (ABP **5a**), β-glucuronidases^31^ (ABP **6a**), and α-iduronidases^44^ (ABP **7a**). As illustrated in Scheme 1, the fluorescent versions of these probes are designated **a**, and biotinylated analogs are denoted as **b**.

**Figure 1:**
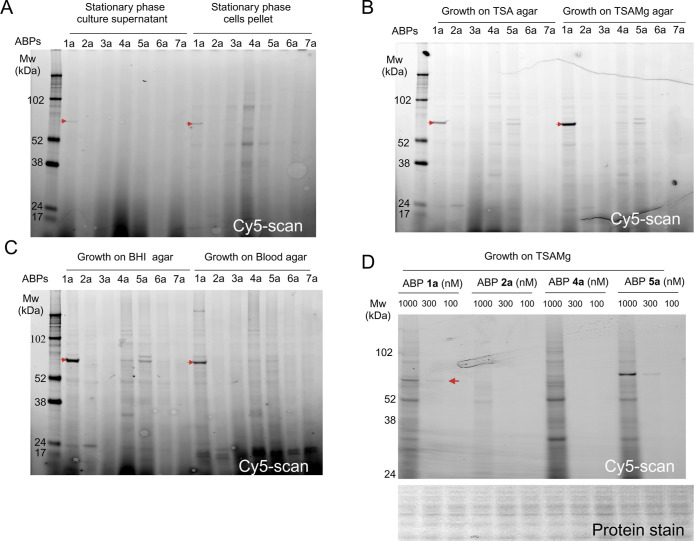
Fluorescent ABP-labeling profiles in *S. aureus* USA300 JE2 using glycosidase ABPs. Live bacteria were subjected to labeling with 1 μM of the ABPs (**1a**–**7a**) for 60 min at 37 °C, followed by cell lysis and subsequent SDS-PAGE analysis. The graph illustrates the results with fluorescent scans captured in the Cy5 channel (635 nm wavelength) using the Amersham Typhoon 5 (cytiva) imaging system. (A) Live cells were grown to stationary phase culture in TSB and fractionated into culture supernatant and cell pellet. (B) Live bacteria were labeled after cultivation on TSA or TSAMg. (C) Labeling profile of *S. aureus* USA300 JE2 live cells, either from grown on BHI agar or blood agar. Red arrowheads indicate the most prominent band labeled by ABP **1** in all conditions. (D) Dose-dependent labeling profile of *S. aureus* USA300 JE2 cells harvested from TSAMg with ABPs **1a**, **2a**, **4a**, and **5a**. The upper panel shows a Cy5-scan. A vertically compressed image of the gel after protein staining (SimplyBlue Safe Stain) is shown in the lower panel below.

As a bacterial model strain, we used JE2, which is a derivative of the clinically relevant methicillin-resistant MRSA lineage USA300 and for which availability of a transposon mutant library^45^ facilitates target validation. Initially, we applied this set of probes to profile active enzymes during liquid culture in TSB. After labeling of live cells, samples were fractionated to separate cell culture supernatant including secreted proteins from cell pellet. Both the supernatant and the cell pellet were lysed prior to SDS-PAGE and in-gel fluorescence scanning. The only ABP returning a fluorescent band in the SDS-PAGE gel from the culture supernatant sample was ABP **1**-Cy5, revealing a putative α-glucosidase with a size of approximately 60–70 kDa (Figure 1A). The same probe also labeled a target protein in the cell pellet (Figure 1A). In the cell pellet, several bands below the 102 kDa marker appeared to be labeled indiscriminately by all fluorescent probes.

We continued to profile retaining GH activity upon growth on agar, testing diverse media including tryptic soy agar (TSA), TSAMg, which promotes biofilm formation,^46^ as well as brain-heart-infusion (BHI) and blood agar, which both contain host-tissue derived factors that may include physiologically more relevant substrates for GH. We observed that the activity profiles revealed by the various probes were rather similar across the different growth media. Under all conditions, the band at 60–70 kDa labeled by ABP **1** was the most prominent band (Figure 1B,C). However, upon growth on agar, ABPs **2a**, **4a**, and **5a** labeled a few unique bands with molecular weights of approximately 24, 102 kDa, and another band in the 60–70 kDa range that ran slightly higher than the one labeled by **1a** (Figure 1B,C). Labeling of probes **1a**, **2a, 4a** and **5a** was dose-dependent, and a concentration of 1 μM proved gave robust labeling (Figure 1D).

**Figure 2:**
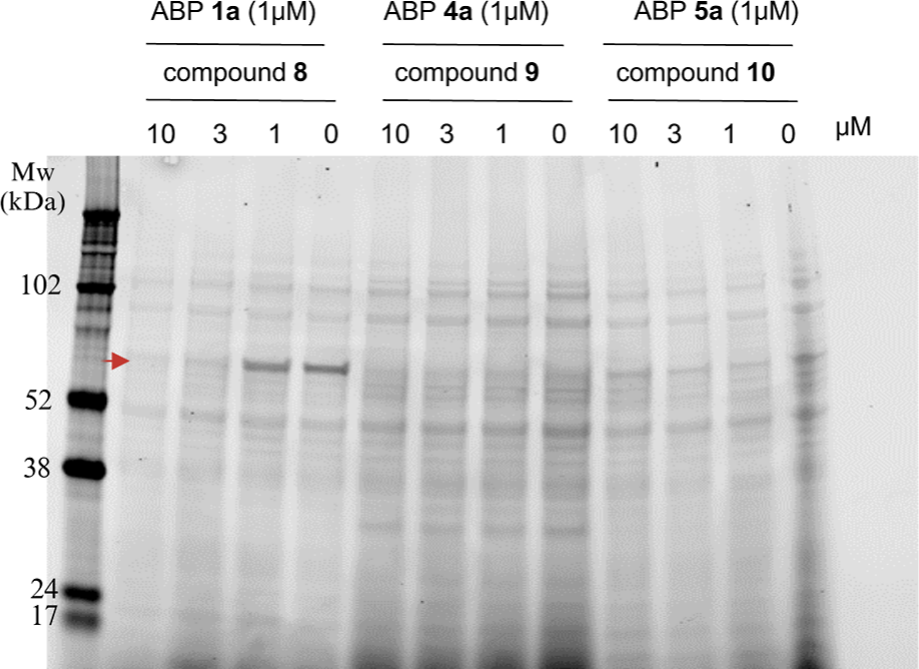
Competitive Activity-Based Protein Profiling (ABPP) in *S. aureus* USA300 JE2. The cells were preincubated with compounds **8**, **9**, and **10** at the indicated concentrations for 60 min before the addition of fluorescent ABPs **1a**, **4a** and **5a**, respectively. The graph shows fluorescent scans captured in the Cy5 (635 nm) channel using the Amersham Typhoon 5 (cytiva) imaging system. Band densitometric analysis for the bands labeled by **1a** and data from another replicate including a protein stain as loading control are shown in Figure S1.

To determine whether the labeling profiles by ABPs **1a**, **4a** and **5a** (on TSAMg) resulted from specific binding to the active site, we examined the effect of preincubation with unlabeled parent inhibitors (compounds **8**, **9** and **10**) in a competitive ABPP experiment (Figures 2 and S1). Preincubation with compound **8** (1–10 μM) resulted in reduced labeling of the most pronounced bands at >55 kDa, suggesting that compound **8** competes with ABP **1a**. In contrast, preincubation with compounds **9** or **10** did not alter the labeling patterns for ABP **4a** and **5a**, respectively, up to a concentration of 10 μM. This outcome could indicate a nonspecific labeling mechanism of ABP **4a** and **5a**, but may also indicate low inhibitor potency.

### Target Identification by Chemoproteomics

To identify putative enzymatic targets of ABPs **1**, **4** and **5** we used biotinylated analogs of these probes for mass-spectrometry based ABPP. In analogy to previous studies^20,21^ bacteria were cultivated on TSAMg, where the fluorescent analogs of these probes gave robust, differential labeling profiles (Figure 1B). Cells were labeled with the pool of probes **1b**, **4b** and **5b** or treated with vehicle (DMSO) as a control, then lysed, and biotinylated proteins were enriched using a streptavidin resin. Enriched, trypsinized samples were then subjected to analysis by liquid chromatography-tandem mass spectrometry (LC–MS/MS).

Ten proteins were significantly enriched in the biotin-ABP-pool treated samples compared to the vehicle-treated control (*p*-value <0.05, enrichment >1.5-fold) (Table 3 and Supporting Information set 1, data are available from ProteomeXchange with identifier PXD052286). Three of these proteins are annotated as putative GH family proteins: 6-phospho-β-glucosidase (BglA), the α-amylase family protein trehalase C (TreC) and autolysin (Atl).

**Table 1:** Bacterial Strains Used in This Study^a^

strain	description	ref/source
*S. aureus* USA300 LAC	wild-type USA300 Los Angeles County (LAC) clone; multilocus sequence type 8, SCCmec type IV cured of antibiotic resistance plasmid	(54)
sgRNA (*atl*)	USA300 LAC carrying pLOW-Plac-dcas9, pVL2336-sgRNA(atl), ery^R^, cm^R^	this study
sgRNA (*bglA*)	USA300 LAC carrying pLOW-Plac-dcas9, pVL2336-sgRNA(bglA), ery^R^, cm^R^	this study
sgRNA (*treC*)	USA300 LAC carrying pLOW-Plac-dcas9, pVL2336-sgRNA(treC), ery^R^, cm^R^	this study
sgRNA (NTC)	USA 300 LAC carrying pLOW-Plac-dcas9, pVL2336-sgRNA(NTC), ery^R^, cm^R^	this study
*S.aureus* USA300 JE2	a plasmid-cured derivative of USA300 LAC and parent strain of nebraska transposon mutant library	(45)
JE2_*atl*: Tn	transposon insertion mutant in JE2 SAUSA300_0955 (*atl*); ery^R^, linc^R^	(45)

a cm^R^, chloramphenicol resistance; ery^R^, erythromycin resistance; lincomycin resistance; linc^R^; NTC, nontargeted control.

**Table 2:** Sequences of sgRNA Base Pairing Regions

targets	sequence 5′ → 3′
*atl*	TTAAATAGCTCTTCTTTCGT
*bglA*	TACAGCTGTAAGAATGTCTT
*treC*	AGGATAAATTTGATATACAA
*Luc* (nontarget control, NTC)	CGGCGCCATTCTATCCTCTA

**Table 3:** LC–MS/MS-Based Target Protein Identification Using a Cocktail of GH-Biotin Probes **1b**, **4b**, and **5b** in *S. aureus* USA300 JE2

accession number	protein	description	gene name	unique peptides	mol weight [kDa]	abundance ratio: (biotin)/(ctrl)	*P*-value
WP_000974460.1	OsmC	organic hydroperoxide resistance protein	*osmC*	2	15.3	3.5	1.97144 × 10^–5^
WP_000159750.1	MurP	PTS transporter subunit EIIC	*murP*	6	50.6	3.3	0.052059494
QPB86698.1	EutD	phosphate acetyltransferase	*eutD*	4	34.9	1.8	4.95726 × 10^–7^
WP_000163988.1	BglA	6-phospho-beta-glucosidase	*bglA*	2	54,7	1,8	1.85817 × 10^–7^
WP_000275730.1	PfbA	pectate lyase	*pfbA*	7	40,7	1,7	0.015415869
QPB87272.1	PlsX	phosphate acyltransferase PlsX	*plsX*	4	35.4	1.5	6.43444 × 10^–10^
WP_000031515.1	TreC	alpha,alpha-phosphotrehalase	*treC*	6	63.5	1.5	5.94054 × 10^–6^
QPB88486.1	GpmA	2,3-diphosphoglycerate-dependent phosphoglycerate mutase	*gpmA*	4	26.7	1.5	4.57359 × 10^–5^
QPB88582.1	Fbp	fructose-1,6-bisphosphatase	*fbp*	4	76.1	1.5	1 × 10^–17^
WP_001074556.1	Atl	glucosaminidase domain-containing protein	*atl*	29	137.3	1.5	0.022711654

### Gel-Based Target Validation

In our previous ABPP studies in *S. aureus* we functionally validated mutants deficient in the identified target genes using mutants from the Nebraska Transposon Mutant Library.^20,21^ However, PCR-based validation of the putative *bgl*A, *tre*C and *atl* transposon mutants (Figure S2) revealed that only the *atl*::Tn mutant had the transposon insertion in the correct gene.

**Figure 3:**
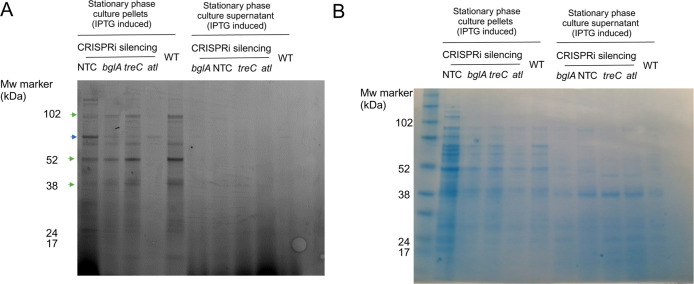
Validation of ABP-labeling profiles using CRISPRi gene-silencing. (A) Labeling profiles of *S. aureus* USA 300 LAC CRISPRi constructs targeting *luc* (nontargeting control NTC) and the GH genes, *atl*, *bglA*, and *treC* with probe **1a** at 1 μM. LAC WT was used as a control. The cells were cultivated in stationary phase culture in TSB supplemented with 250 μM IPTG to induce dCas9 expression and fractionated into a cell pellet (left panel) and culture supernatant (right panel). Fluorescence scan was captured in the Cy5 (635 nm) channel using the Amersham Typhoon 5 (cytiva) imaging system. Green arrowheads indicate bands that are absence in the *atl*-silencing strain. The blue arrowhead indicates the dominant 60–70 kDa band. (B) Protein stain (simply blue safe stain) of the same gel shown in A.

As an alternative way to evaluate the function of BglA, TreC and Atl, we generated IPTG-inducible CRISPRi interference strains in the MRSA strain USA300 LAC, allowing for conditional knock-down of the *tre*C, *bgl*A and *atl* genes, respectively. We compared the labeling profiles of probe **1a** in stationary phase cell-pellets from the IPTG-induced CRISPRi constructs compared to those of a nontarget control (NTC), where the sgRNA targets a gene sequence derived from the luciferase (*luc*) gene (Figure 3A,B). Of note, the bands at approximately 120 kDa, 52 kDa and 38 kDa disappeared after silencing of *atl* (Figure 3A, green arrows). Full length autolysin has a molecular weight of 137 kDa and a 51 kDa fragment has been reported as its cleaved glucosaminidase domain.^10^ Similar changes to the labeling profile were seen for the *atl* transposon mutant (Figure S3) confirming result from the CRISPRi knock-down.

Probe **1a** also labeled a protein with a size of 60–70 kDa in cell-pellet (Figure 3A, blue arrow). Although this size matches the predicted molecular weight of TreC (63.5 kDa), the band was still detected after silencing of *tre*C (Figure 3A,B). Neither of the bands labeled by probes **4a** and **5a** in stationary phase cells disappeared in IPTG-induced CRISPRi constructs (Figure S4) supporting the notion that the labeling profiles of these probes might be rather nonspecific.

### Functional Role of Identified Glycosidase Hydrolases

**Figure 4:**
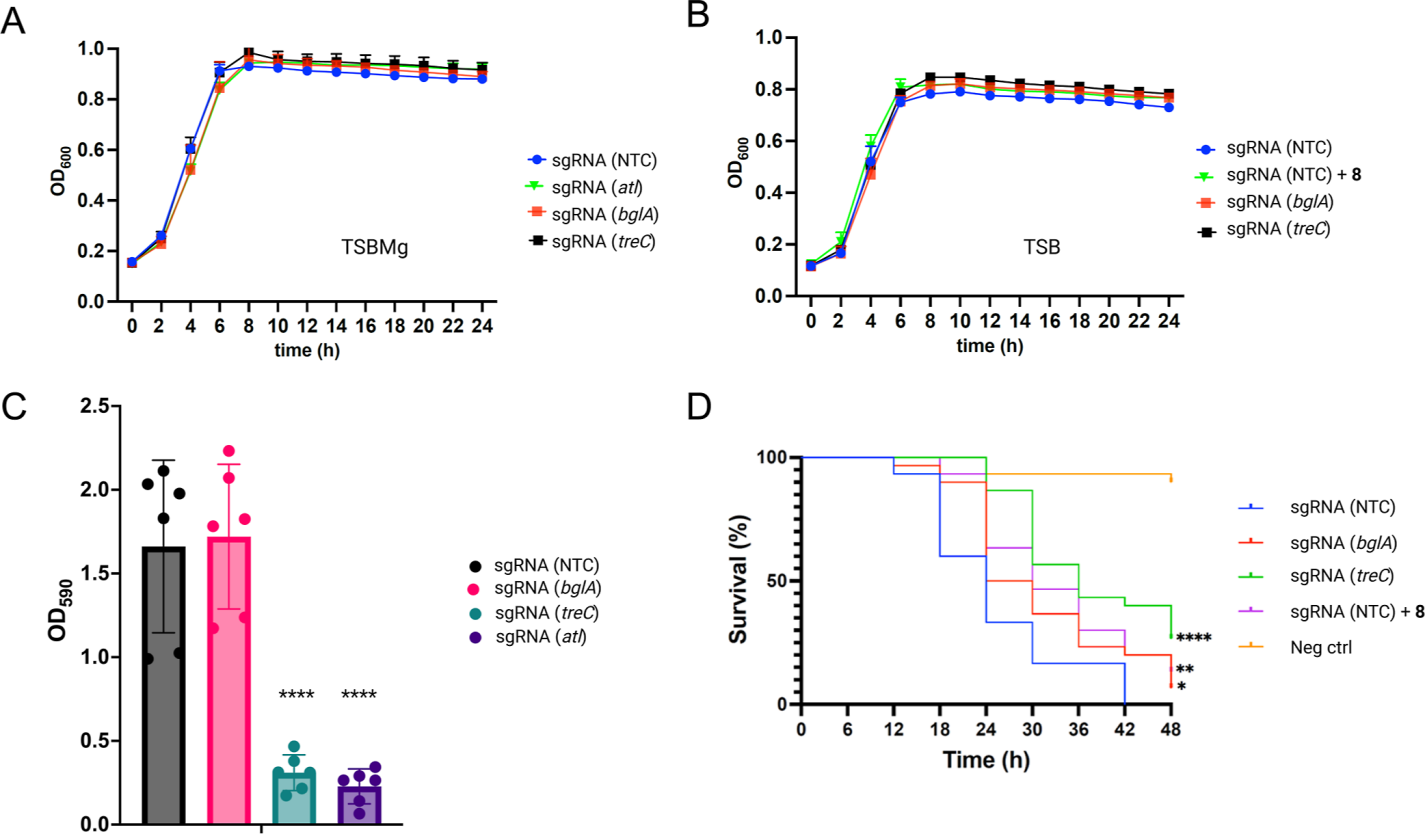
Functional validation of *S. aureus* CRISPRi knockdown strains. (A) Growth curves of *S. aureus* CRISPRi constructs (with the indicated sgRNAs) including nontargeting control (NTC) sgRNA in TSBMg supplemented with 250 μM IPTG. The curves show means ± standard deviation of *n* = 3 independent biological replicates. (B) Growth curves of *S. aureus* CRISPRi constructs, including nontargeting control (NTC) sgRNA and nontargeting control (NTC) sgRNA treated with 3 μM compound **8**, measured in TSB supplemented with 250 μM IPTG. The curves show means ± standard deviation of *n* = 3 independent biological replicates. (C) Biofilm formation of CRISPRi constructs. The graph shows means ± standard deviation of six independent biological culture replicates (derived from 4 technical replicates each) that were pooled from two independent experiments. Dunnett’s one-way ANOVA was used for comparison to the nontargeting control (NTC) sgRNA (****p < 0.0001, ns indicates no significant difference). (D) Kaplan–Meier (KM) survival plots of *G. mellonella* larvae after inoculation with *S. aureus* nontargeting control (NTC) sgRNA, CRISPRi strains, and nontargeting control (NTC) sgRNA treated with 3 μM compound **8**. Precultures were grown overnight with 250 μM IPTG to induce dCas9 expression and silencing of the target gene. The plots indicate the average of *n* = 3 independent biological replicates (i.e., bacterial cultures originating from 3 different colonies), that were each used to infect 10 larvae per group. Mortality was monitored every 6 h for 48 h (*N* = 180). Larvae injected with PBS served as a negative control. The asterisks in the graph indicates significant difference in survival of IPTG-induced *treC*, *bgl*A, compared to nontargeting control (NTC) sgRNA (*p < 0.05, **p < 0.01, ****p < 0.0001), as determined by the log-rank (Mantel–Cox) test (*p* < 0.0001).

Finally, we aimed to determine whether the newly identified GHs TreC and BglA are relevant for bacterial biofilm formation or virulence. First, we compared the growth of the IPTG-inducible CRISPRi-strains in TSBMg or TSB. Regardless of the presence of IPTG to induce dCas9 expression, constructs targeting *bgl*A, *tre*C or *atl* all showed similar growth curves compared to nontargeted control (NTC), suggesting that silencing of these genes does not affect *in vitro* growth in TSBMg or TSB (Figure 4A,B). Treatment of the NTC with compound **8**, which competed away TreC labeling by ABP **1a** in our competitive ABPP results (Figure 2), did not affect growth in TSB either (Figure 4B). We then assessed the biofilm-forming ability of the newly identified TreC and BglA using a crystal violet assay (Figure 4C). Given that autolysin has already been shown to contribute to *S. aureus* biofilm formation,^47^ we also included a CRISPRi construct targeting the *atl* gene as a control. This experiment revealed that silencing of *treC*, but not *bglA* exhibited pronounced reduction in biofilm formation compared to the nontargeted control (Figure 4C). As expected, silencing of *atl* also resulted in reduced biofilm formation (Figure 4C).

Subsequently, we used a *G. mellonella* infection model to test whether these enzymes contribute to the virulence of *S. aureus*. We treated the corresponding *S. aureus* CRISPRi strains with IPTG *in vitro* to silence *tre*C, *bgl*A or nontargeted control (NTC), and used these strains to infect *G. mellonella* larvae. Survival times of the infected larvae were compared with those of PBS-inoculated larvae (negative control) for 48 h. Larvae infected with the induced CRISPRi constructs for *bgl*A, *tre*C and nontargeted control (NTC) expired more rapidly than PBS-inoculated larvae, indicating that carriage of the CRISPRi plasmids infers a fitness cost in this infection model (Figure 4D). However, larvae infected with nontargeted control (NTC) *S. aureus* died within 42 h (Figure 4D), while larvae that were infected with *tre*C-silenced *S. aureus*, had significantly higher survival rate with approximately 30% of the larvae remaining alive after 48 h (*p* < 0.0001) (Figure 4D). Silencing of *bgl*A had a minor, but statistically significant positive effect on the survival of the larvae when compared to larvae infected with the NTC strain (Figure 4D)

Finally, pretreatment of nontargeted control (NTC) CRISPRi *S. aureus* with the putative TreC inhibitor **8** at a concentration of 3 μM, also significantly increased survival of the larvae (*p* < 0.01). These data establish a role for the previously uncharacterized α-amylase family protein, TreC, in the virulence and biofilm formation of *S. aureus*.

### Conclusions

Here, we present the first ABPP study on identification of retaining GHs in a bacterial pathogen. Since GHs have a high degree of substrate selectivity and no broad-spectrum probe is available, we first profiled a set of 7 different GH-reactive ABPs by gel-based ABPP and then used biotinylated analogs of the most promising probe candidates in probe cocktail-based strategy^48^ for MS-based target identification. Using a cocktail of probes with selectivity for α-glucosidases (ABP **1b**), β-galactosidases (ABP **4b**) and α-fucosidases (ABP **5b**) we identified three putative glycosidases; the putative α-amylase family protein phosphotrehalase (TreC), 6-phospho-β-glucosidase (BglA), and the previously characterized autolysin (Atl).^11−13^ The annotation of these enzymes in combination with experimental validation using CRISPRi mutants suggest that these targets have been enriched predominantly by cyclophellitol-aziridine ABP **1b**.

Since the probe **1** is based on an α-configurated cyclophellitiol aziridine inhibitor known to target retaining α-glucosidases,^38^ the annotation of TreC as an α-amylase family protein makes it an expected target for ABP **1**. Since the parent inhibitor cyclophellitol is also known to interact with beta-glycosidases (although with lower affinity^38^), it seems plausible that the β-glucosidase BglA and β-glucosaminidase Atl have been enriched through ABP **1b**. The expected molecular weight of TreC 63.5 kDa matches the size of the dominant band labeled by probe **1a** upon growth on agar and its labeling was competitively reduced by pretreatment with compound **8**. A much weaker band of similar size was also observed in stationary phase cells. However, gel-based ABPP experiments using CRISPRi silencing under these conditions did not confirm the identity of this band as either TreC or BglA and we conclude that it may correspond either to an unidentified GH, or it could represent a combination of TreC with BglA and/or other unidentified proteins. One potential candidate for this band could be the 58 kDa α-amylase reported previously.^49^ However, this enzyme was not enriched in the MS-data set, which we hypothesize could be due to a lack of expression or activity upon agar-based growth. Unfortunately, technical challenges in maintaining IPTG-levels necessary for dCas9-induced gene silencing prevented us from assessing the labeling profiles of CRISPRi strains under these conditions. We believe that the difference in growth conditions used for the proteomics experiment (agar-based growth) and those compatible with validation of CRISPRi strains (stationary phase, liquid culture) could be one explanation for our inability to assign the major band identified by gel-based ABPP.

Of note, several fainter bands labeled by ABP **1a** disappeared after silencing of *atl*, suggesting they may be fragments of this peptidoglycan hydrolase. The 51 kDa band matches the size reported for the cleaved glucosaminidase domain of autolysin.^10^ However, since labeling was performed in live cells, it also remains possible that the lack of the autolysin reduces cell wall permeability of the probe and thus indirectly affects labeling of other proteins. Heterologous expression of these enzymes will be necessary to unambiguously address the question of their reactivity of the α-cyclophellitol aziridine-ABP **1**, and a biochemical characterization of both BglA and TreC is necessary to assess whether their current functional annotation is correct. For the β-galactosidase probe ABP **4a** and the α-fucosidase probe ABP **5a**, we conclude that the gel-based labeling patterns are most likely nonspecific. This notion is supported by competitive ABPP with unlabeled inhibitors that did not give evidence for a specific active site-directed binding that would lead to competition with the probe.

Besides the three putative GHs discussed above, our MS-based ABPP data set also included 7 additional proteins that were enriched after treatment with the probe cocktail. Some of these proteins are hydrolytic enzymes (phosphate acyltransferase and phosphate acetyltransferase) which might have active site residues reactive to the aziridine electrophile, whereas others seem to have an affinity for sugars (pectate lyase, fructose-1,6-bisphosphatase and the sugar phosphotransferase system *mur*P). The molecular basis for these interactions remains to be determined.

Functional validation of the newly identified enzymes using CRISPRi gene silencing revealed that TreC, but not BglA, contributes to bacterial biofilm formation on plastic surface in a crystal violet assay. TreC also contributes to bacterial virulence in a *G. mellonella* model, whereas silencing of neither gene affected growth in regular rich media. Since the application of IPTG in the wax moth larvae *in vivo* is challenging, ITPG was already added to induce dCas9 expression (and knock down *tre*C) during the preculture period before infection. Bacteria may overcome this knock-down through *de novo* expression of *tre*C, during the 48 h experiment, suggesting TreC is functionally important early in the infection process.

Intriguingly, application of compound **8**, an α-configured cyclophellitol-aziridine which is the parent compound of ABP **1**, also attenuated virulence of WT strains. Compound **8** was previously shown to be a potent inhibitor of human lysosomal glucosidase^38^ and here, we show that in live *S. aureus* cultures it specifically competes with bands labeled by ABP**1a** (even though these could not be unambiguously assigned to a single GH). Whereas biochemical studies are needed to confirm that compound **8** indeed inhibits TreC and the physiological substrates of this enzyme remain to be identified, our data suggest that this enzyme is a viable antivirulence target candidate.

Of note, we performed chemoproteomic profiling under simplistic *in vitro* conditions in which probably not all infection-relevant glycosidase enzymes are expressed. Similar profiling studies performed in more complex models for infection, host-interaction and microbe–microbe interactions may identify additional relevant, uncharacterized enzymes that could act on substrates derived from host factors or microbial competitors.

## Experimental Section

### Bacterial Strains and Culture Conditions

This study utilized *S. aureus* strains USA300 LAC, USA300 JE2, and their respective isogenic mutants, as summarized in Table 1. All strains were cultured routinely on tryptic soy agar (TSA), blood agar, brain-heart-infusion (BHI) agar, TSA supplemented with 100 mM MgCl_2_ (TSAMg), or in difco tryptic soy broth (TSB) or TSB supplemented with 100 mM MgCl_2_ (TSBMg). Incubation of all bacterial strains was carried out at 37 °C, and liquid cultures were aerated by shaking at 180 rpm unless specified otherwise.

### Probes and Inhibitors

All probes and inhibitors were synthesized as described previously. The following ABPs were used: **1a**/**b**,^38^**2a**,^39^**3a**,^40^**4a/b**,^28^**5a/b**,^42,43^**6a**,^31^**7a**.^44^ Cy5-labeled versions of the probes are referred to as **a**, biotin-labeled analogs as **b**. The following nontagged inhibitors were used: **8**,^38^**9**^28^ and **10**.^44^

### Labeling with Fluorescent Activity-Based Probes

Bacterial cultures grown overnight on agar plates or in liquid culture were adjusted to the desired cell density in TSB and transferred to microtubes, with a final volume of 50–100 μL. GH ABPs were then added (from 100× stock solutions in DMSO) to a final concentration of 1 μM and the cells were incubated for an additional 60 min at 37 °C and 300 rpm. For the competitive ABPP assays, compounds **8** (Chris 021), **9** (TB562), or **10** (JJB330) were added from 100× concentrated stock solutions in DMSO to bacterial cultures to achieve final concentrations of 1, 3, or 10 μM, respectively. Samples were preincubated for 60 min at 37 °C with shaking at 300 rpm prior to ABP treatment. Following ABP treatment, bacterial suspensions were transferred to 2 mL screw-cap tubes containing 30–50 μL of 4× SDS-loading buffer (40% glycerol, 240 mM Tris/HCl at pH 6.8, 8% SDS, 0.04% bromophenol blue, and 5% β-mercaptoethanol) and approximately 60–100 μL of 0.1 mm glass beads, and the bacteria were lysed by bead-beating.

### SDS-PAGE Analysis of Fluorescently Labeled Proteins

Upon addition of the 4× SDS sample buffer, the probe-labeled bacterial lysates were boiled at 95 °C for 10 min and separated by SDS-PAGE. The resulting wet gel slabs were scanned for fluorescence in the Cy5 (635 nm) and Cy2 (488 nm) channels, utilizing the Amersham Typhoon 5 (cytiva) imaging system. For protein staining, the gel was washed three times with ultrapure water to remove excess buffer. SimplyBlue SafeStain (Thermo Fisher Scientific) was added, and the gel was incubated for 1 h at room temperature with gentle rocking. Following staining, the gel was rinsed with ultrapure water for 1 h to remove excess stain and then left in water overnight for optimal background clearance. Protein bands were imaged using a gel imaging system. Band densitometric analysis was performed with Fiji version 2.9.0.

### Labeling with Biotinylated Probes and Sample Preparation for Mass-Spectrometry

*S. aureus* USA300 JE2 cultures were cultivated on TSAMg plates for 24 h and resuspended to an approximate OD_600_ of 20 in 3 mL of TSB. For each biological replicates (R1–R3), 1 mL aliquots were transferred to 1.5 mL tubes, and either a biotin-tagged ABP (2 μM) or DMSO as control was added. The cells were incubated for 60 min at 37 °C and 700 rpm before samples were centrifuged at 4500*g* for 5 min at 4 °C, after which the supernatant was removed. After the incubation, the samples were centrifuged at 4500*g* for 5 min at 4 °C, and the supernatant was carefully removed. The samples were centrifuged at 4500*g* for 5 min at 4 °C, and the supernatant was carefully removed. The cell pellets were resuspended in 1.2 mL of RIPA Lysis buffer (50 mM Tris, 150 mM NaCl, 0.1% SDS, 0.5% sodium deoxycholate, 1% Triton X-100) and lysed by bead-beating. Samples were centrifuged for 5 min at 10,000*g* at 4 °C, and the protein concentration in the supernatant was adjusted to 1.0 mg/mL. Proteins were subsequently stored at −20 °C until sample preparation.

For each sample, 50 μL of streptavidin magnetic beads (Thermo Fisher, 88817) were washed twice with 1 mL of RIPA lysis buffer, and the beads were then incubated with 1 mg of protein from each sample in an additional 500 μL of RIPA lysis buffer at 4 °C overnight on a rotator with an 18 rpm speed. After enrichment, the beads were pelleted using a magnetic rack, and washed twice with RIPA lysis buffer (1 mL, 2 min at room temperature), once with 1 M KCl (1 mL, 2 min at room temperature), once with 0.1 M Na_2_CO_3_ (1 mL, ∼10 s), once with 2 M urea in 10 mM Tris–HCl (pH 8.0) (1 mL, ∼10 s), and twice with RIPA lysis buffer (1 mL per wash, 2 min at room temperature). After the final wash, the beads were transferred to fresh protein Lo-Bind tubes with 1 mL of RIPA lysis buffer. The beads were then washed three times with 500 μL of 4 M urea in 50 mM ammonium bicarbonate (Ambic) with shaking for 7 min each time to remove nonspecifically enriched proteins. Finally, the beads were washed three times with 500 μL of 50 mM Ambic with shaking for 7 min, changing the tube between these washes.

For on-bead digestion, 150 μL of 50 mM Ambic, 3 μL of 1 mM CaCl_2_, 0.75 μL of 1 M dithiothreitol (DTT), 4.5 μL of 500 mM iodoacetamide (IAA), and 6 μL of MS-grade trypsin solution were added to the protein Lo-Bind tube. The samples were incubated at 37 °C overnight on a shaker at 800 rpm. After overnight incubation, samples were spun down, and the supernatant containing the tryptic digests was collected, and the remaining beads were washed with 70 μL of 50 mM Ambic. For each sample, 20 μL of formic acid were added to the combined eluates, and the samples were then stored at −20 °C until their analysis via LC–MS/MS.

### Liquid Chromatography–Mass Spectrometry Analysis

Varian’s OMIX C18 tips were used for sample cleanup and concentration. Peptide mixtures containing 0.1% formic acid were loaded onto a Thermo Fisher Scientific EASY-nLC1200 system equipped with a C18 column (2 μm, 100 Å, 50 μm, 50 cm) and subjected to fractionation using a 5–80% acetonitrile gradient in 0.1% formic acid at a flow rate of 300 nL/min for 60 min. The separated peptides were analyzed on a Thermo Scientific Orbitrap Exploris 480 instrument, employing a data-dependent mode with a Top20 method. Raw data was processed using Proteome Discoverer 2.5 software, and fragmentation spectra were searched against the *S. aureus* 300 LAC database. The search employed peptide mass tolerances of 10 ppm and a fragment mass tolerance of 0.02 Da. A false discovery rate (FDR) of 5% was applied for peptide identification. To ensure accuracy, the filter criterion of two unique peptides was used, and three replicates were performed for each sample. Protein abundance obtained from Proteome Discoverer was averaged across replicates, and the ratio of the ABPP-enriched sample versus mock-treated control sample was calculated. In cases where missing values occurred across all replicates for a given condition, a value of 1 was assigned to facilitate downstream ratio calculation. Proteins enriched more than 1.5-fold by the biotinylated probe were selected. Statistical significance (*p*-values) was determined using the two-tailed *t*-test implemented within Proteome Discoverer 2.5, and a Benjamini–Hochberg correction was applied to control the false discovery rate (FDR), ensuring that the results reflect genuine differences in protein abundance. Proteins were considered statistically significant if they met the adjusted *p*-value threshold of <0.05, as determined by the statistical framework implemented in Proteome Discoverer. While performing ABPP-enriched and other pull-down experiments, enrichment of nonspecific binding is a limitation.^50,51^ To minimize false positives, any ribosomal proteins, proteins with known affinity for hydrophilic beads, as well as endogenously biotinylated proteins (e.g., carboxylase family proteins) that interact with streptavidin-beads regardless of ABP-labeling, were excluded from the data set. The mass spectrometry proteomics data have been deposited to the ProteomeXchange Consortium via the PRIDE^52^ partner repository with the data set identifier PXD052286.

### Construction of *S. aureus* CRISPRi Strains

The CRISPRi system in *S. aureus* includes two plasmids. One plasmid harbors the dCas9 under the control of an IPTG-inducible promoter (P*lac*), and a second plasmid (pVL2336) that constitutively expresses the sgRNA.^53^ To construct CRISPRi strains in *S. aureus* USA 300 LAC, the bacteria were first equipped with the plasmid carrying P*lac*-dCas9. The pVL2336 plasmid is designed to facilitate the insertion of gene-specific sgRNAs through Golden Gate cloning using the *Bsm*BI restriction enzyme. The sgRNA oligonucleotides are composed of a 20-nucleotide (nt) sequence that pairs with the nontemplate DNA strand of the target gene near the 5′ end and adjacent to a PAM (Protospacer Adjacent Motif) sequence. These oligonucleotides also include a 4-base pair (bp) overhang that matches the overhang region generated by *Bsm*BI digestion. The resulting sgRNA plasmids then allow for the expression of sgRNAs that consist of a 20 bp gene-specific sequence, the Cas9 handle, and a transcriptional terminator under the control of a constitutive promoter.^53^ The plasmid pVL2336 was digested using restriction enzyme *Bsm*BI and then ligated with sgRNA oligonucleotides (Table 2) using T4 DNA ligase. sgRNA oligonucleotides were derived from *treC*, *bglA* and *atl* as well as the luciferase gene (*luc*) as a nontargeted control (NTC). The assembled plasmids were then transformed into *E. coli* IM08B and, following that, into *S. aureus* LAC. To identify successful transformants, selection was initially carried out using plates containing erythromycin (5 μg/mL) and chloramphenicol (10 μg/mL) antibiotics, followed by verification through PCR and sequencing.

### PCR Validation of Transposon Mutants

To verify the transposon mutants a PCR was performed using the Phire Hot Start II PCR Master Mix (Thermo Scientific, USA). The PCR was performed according to the manufacturer protocol using the primers *bglA* F–R, treC F–R and *atl* F–R (Table S1) to amplify the *bglA*, *treC* and *atl* fragments respectively. The *S. aureus* USA 300 JE2 (WT) was amplified as a positive control. The thermal profile was generated in a thermal cycler (BioRad, USA) and included an initial heat-denaturing step at 95 °C for 30 s; 35 cycles at 98 °C for 5 s, 60 °C for 5 s, 72 °C for 15 s and a final extension at 72 °C for 1 min. Amplified products were analyzed by electrophoresis in a 1% agarose gel stained with GelRed Nucleic acid stain (Biotium, USA).

### Growth Curve

Growth curves were generated using 96-well microtiter plates. Cultures grown overnight were diluted in fresh medium at a 1:100 ratio, and 200 μL of the diluted culture was transferred to each well of the 96-well plate. The plates were then incubated at 37 °C, with the optical density at 600 nm (OD_600_) being recorded every 10 min using a Synergy H1 Hybrid Reader (BioTek) instrument. When necessary, antibiotics erythromycin (5 μg/mL) and chloramphenicol (10 μg/mL) were used for selection purposes. To induce dCas9 expression, 250 μM IPTG was added.

### Biofilm Assay

The CRISPRi strains of *S. aureus* were grown in tryptic soy broth (TSB) supplemented with erythromycin (5 μg/mL) and chloramphenicol (10 μg/mL). The overnight cultures were diluted 1:100 into fresh TSB containing 100 mM MgCl_2_ (TSBMg) and the antibiotics erythromycin (5 μg/mL) and chloramphenicol (10 μg/mL). To induce the expression of dCas9, 250 μM IPTG was added. Subsequently, 200 μL of these cultures were transferred into 96-well plates and incubated overnight at 37 °C without shaking. Postincubation, the wells were washed to remove planktonic cells, and the plates were air-dried. Each well was then stained with 0.1% (wt/vol) crystal violet, sufficient to cover the well surface, and left for 10 min at room temperature. After staining, the wells were washed to remove excess dye and blotted to dry. Each well was then filled with 200 μL of 96% ethanol and left for 10 min at room temperature to solubilize the dye. The absorbance of the solubilized dye was measured at 590 nm. The OD590 values were recorded from 6 biological replicates, each derived from four technical replicates, and were averaged and corrected using blank wells containing only medium.

### Infection of *S. aureus* in *G. mellonella* Larvae

Larvae of *G. mellonella* were sourced from Reptilutstyr AS (Norway). For the infection experiments, *S. aureus* CRISPRi strains were cultured overnight in TSB, supplemented with the necessary antibiotics. For the depletion of the gene of interest, CRISPRi strains were grown with the addition of 250 μM IPTG. After overnight incubation, the bacterial cultures were washed with PBS and then resuspended in PBS to achieve a concentration of 1.0 × 10^8^ cells/mL, followed by further diluted in PBS to reach a final concentration of 10^5^ cells/mL. In the case of inhibitor treatment, the nontargeted (NTC) control sgRNA strain was treated with compound **8** (3 μM) for 1 h prior to infection. For infecting the larvae, 10 μL of this bacterial suspension was injected into the right hind proleg using a 30G syringe microapplicator (0.30 mm (30G) × 8 mm, BD Micro-Fine Demi). A control group of 10 larvae received 10 μL of PBS. The larvae were then placed in 9.2 cm Petri dishes, incubated at 37 °C in darkness, and survival was monitored for 48 h. Each experiment was independently replicated at least three times.

### Statistical Analysis

Graphs and statistical analyses were produced using GraphPad Prism 9. Unless specified otherwise, all experiments were performed using three independent biological replicates. Data for graphs are shown as mean ± standard deviation (SD). To compare nontargeted control (NTC) sgRNA to CRISPRi constructs, one-way ANOVA tests were utilized, followed by Dunnett’s multiple comparisons test, using the NTC sgRNA strain as the control. Significance levels were denoted as follows: *P < 0.05, **P < 0.01, and ****P < 0.0001.

## Supporting Information Available

List of proteins identified by MS-ABPP (Supporting Information set 1) (XLSX)
List of primer sequences used in this study (Table S1); Band densitometry analysis of gel-based competitive ABPP (Figure S1); PCR validation of transposon mutants (Figure S2); Validation of ABP-labeling profiles using transposon mutants (Figure S3); Validation of ABP-labeling profiles using CRISPRi gene-silencing (Figure S4) (PDF)
