# Supplementary material for: Activity-Based Protein Profiling Identifies an α-Amylase Family Protein Contributing to the Virulence of Methicillin-Resistant Staphylococcus aureus
Source: ACS Infect Dis. 2025 Feb 7;11(3):573–83. doi: 10.1021/acsinfecdis.4c00638 (PMC11915364; doi:10.1021/acsinfecdis.4c00638)
Supplement: Supplementary file 2 — id4c00638_si_002.pdf [file id4c00638_si_002.pdf]

Supporting Information

Activity-Based Protein Profiling Identifies an α-amylase Family Protein Contributing to the Virulence of Methicillin-Resistant *Staphylococcus aureus*

Md Jalal Uddin[1], Kjersti Julin[1], Herman S. Overkleeft[2], Mona Johannessen[1] and Christian S. Lentz[1*]

1. Centre for New Antibacterial Strategies (CANS) and Research Group for Host-Microbe Interactions, Department of Medical Biology (IMB), UiT—The Arctic University of Norway, 9019 Tromsø, Norway
2. Leiden Institute of Chemistry, Leiden University, Einsteinweg 55, 2333 CC Leiden, The Netherlands

*To whom correspondence should be addressed: Christian S. Lentz, Christian.s.lentz@uit.no

Table of Contents

Table S1. List of primer sequences used in this study……………………… page S-2

Figure S1. Band densitometry analysis of gel-based competitive ABPP…… page S-3

Figures S2. PCR validation of transposon mutants ……………………. ….. page S-4

Figure S3. Validation of ABP-labelling profiles using transposon mutants… page S-4

Figure S4. Validation of ABP-labelling profiles using CRISPRi gene-silencing page S-5

Supplementary tables

Table S1. List of primer sequences used in this study

Targets	Sequence
bglA F	GCCAGTGCTTTAGCAGTTCG
bglA R	TCTGGATCAATCGCCCAACC
treC F	ACACAACCAGAACGCCAAGA
treC R	TCGCTAACACATCCGCTTCA
atl F	TTATCTTGGGGTGTCGGTGC
atl R	GGTTTCGACGGTGTTGTTGG

Supplementary figures

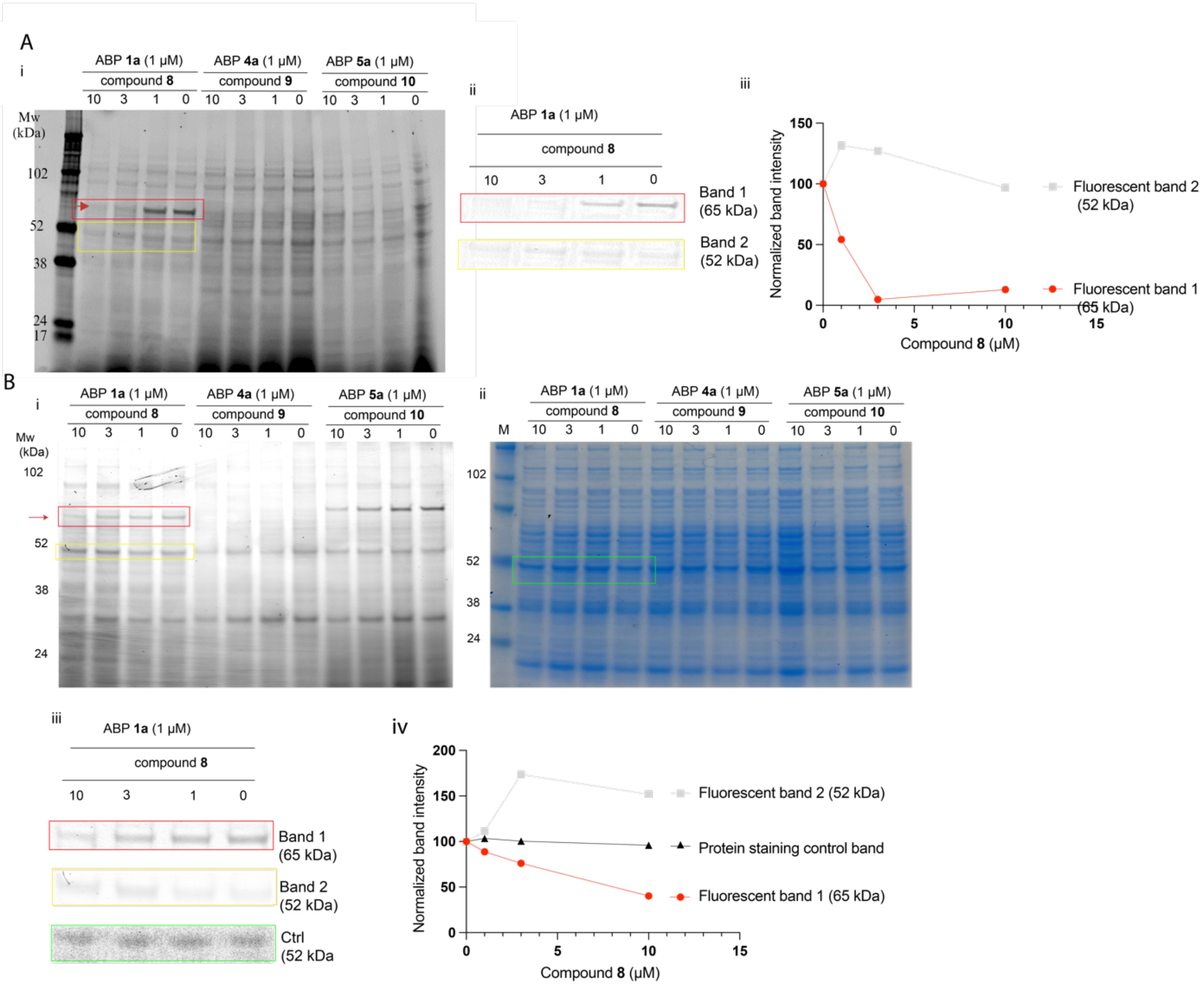

Supplementary Figure S1. Band densitometry analysis of gel-based competitive ABPP. **A)** i) The same gel as shown in figure 2. Here, boxes indicate areas subjected to band densitometry analysis. The figure shows a Cy5-fluorescence scan showing competition of probes **1a**, **4a**, and **5a**, with their respective unlabelled parent inhibitors compound **8**, **9** and **10.** ii) The boxed areas including band 1 (65 kDa, red box) and band 2 (52 kDa, yellow box) labelled by **1a** were subjected to background subtraction (Light background, rolling ball radius: 50.0 – 100.0 pixels) using Fiji version 2.9.0 iii) Normalized band density plotted against the concentration of compound **8** used for preincubation. For each band, the intensity is displayed as percentage value relative to the intensity of the control sample without inhibitor which was set to 100%. **B)** Results from an independent replicate showing i) the ABP-labelling profile (Cy5-scan) and ii) a protein stain as a loading control. iii) The boxed areas were subjected to background subtraction (Light background, rolling ball radius: 50.0 pixels) and individual band density was determined. iv) Normalized band intensity of the bands shown in Biii. For each band, the intensity is displayed as percentage value relative to the intensity of the control sample without inhibitor which was set to 100%.

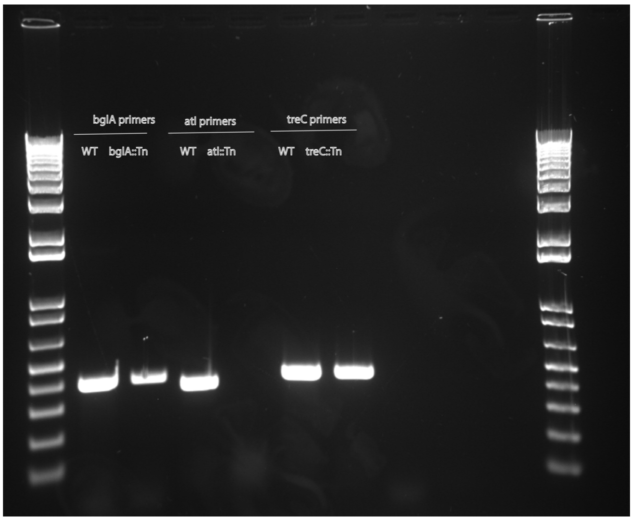

Supplementary Figure S2. **PCR validation of transposon mutants.**
PCR analysis of putative *bgl*A::Tn, *atl*::Tn and *tre*C::Tn transposon mutants. The *bgl*A, *atl* and *tre*C genes were PCR amplified with the corresponding gene-specific primers, samples were analysed by agarose gel electrophoresis and stained with GelRed Nucleic acid stain and visualized by UV-light. The presence of a band indicates the presence of the intact gene, while the absence indicates correct insertion of the transposon.

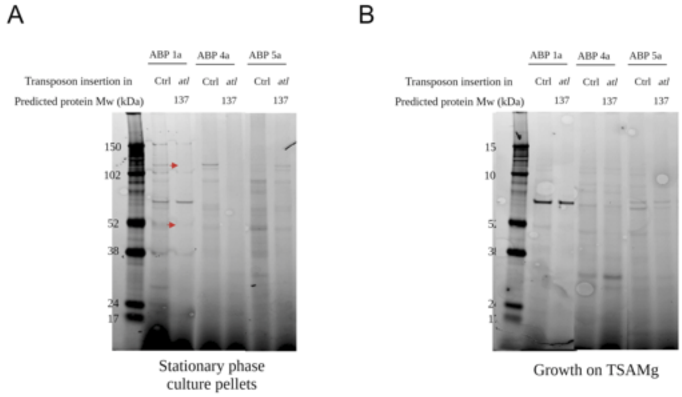

Supplementary Figure S3. **Validation of ABP-labelling profiles using transposon mutants**. A, B) Labelling profiles of *S. aureus* USA 300 JE2 wildtype (Ctrl) and transposon mutant strain with insertion of GH gene, *atl*. Predicted size of full-length Atl: 137 kDa. The bacterial cells were labelled with 1 µM of probes 1a, 4a, or 5a under two different conditions: A) cells grown to stationary phase culture in TSB, followed by fractionation into cell pellets, and B) The cells were harvested in TSAMg. Arrowheads indicate absence or reduction in band intensity in individual mutant strains.

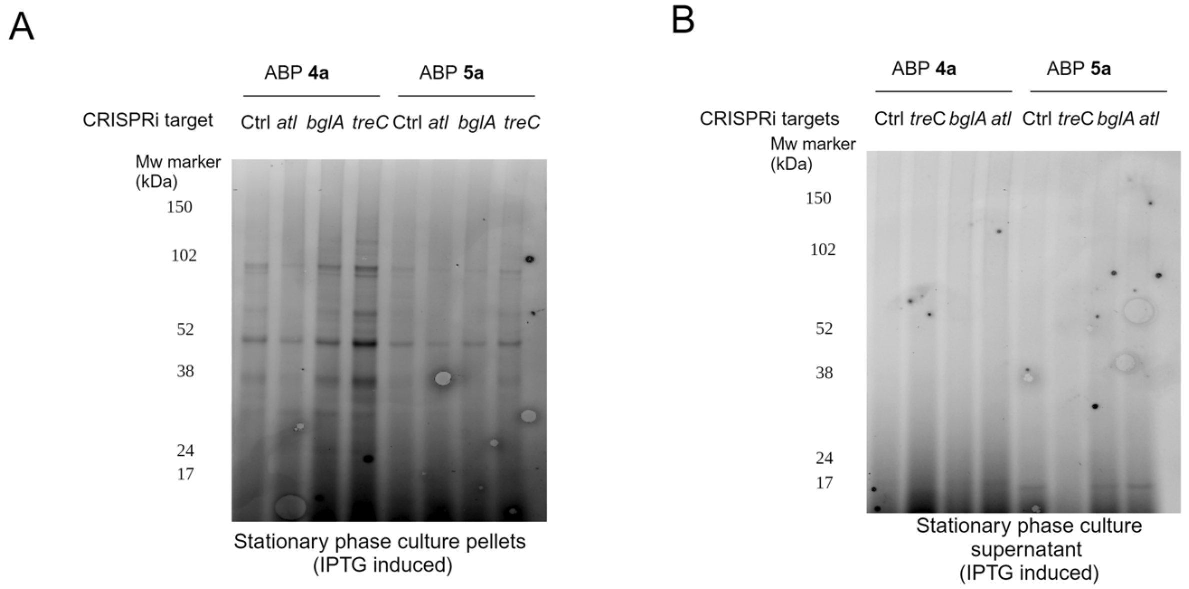

Supplementary Figure S4. **Validation of ABP-labelling profiles using CRISPRi gene-silencing. A**, **B)** Labeling profiles of *S. aureus* USA 300 LAC CRISPRi constructs targeting the GH genes, *atl*, *bglA*, and *treC* with indicated probes at 1 µM. As a control (ctrl) strain LAC WT was used. The cells were cultivated in stationary phase culture in TSB supplemented with 250 µM IPTG to induce dCas9 expression and fractionated into a cell pellet (**A**) and culture supernatant (**B**).
